# Comparative validation of automated presurgical tractography based on constrained spherical deconvolution and diffusion tensor imaging with direct electrical stimulation

**DOI:** 10.1002/hbm.26662

**Published:** 2024-04-22

**Authors:** Ahmed Mohamed Radwan, Louise Emsell, Kristof Vansteelandt, Evy Cleeren, Ronald Peeters, Steven De Vleeschouwer, Tom Theys, Patrick Dupont, Stefan Sunaert

**Affiliations:** ^1^ KU Leuven, Department of Imaging and Pathology Translational MRI Leuven Belgium; ^2^ KU Leuven, Leuven Brain Institute (LBI), Department of Neurosciences Leuven Belgium; ^3^ KU Leuven, Department of Neurosciences, Neuropsychiatry Leuven Belgium; ^4^ KU Leuven, Department of Geriatric Psychiatry University Psychiatric Center (UPC) Leuven Belgium; ^5^ UZ Leuven, Department of Neurology Leuven Belgium; ^6^ UZ Leuven, Department of Neurosurgery Leuven Belgium; ^7^ UZ Leuven, Department of Radiology Leuven Belgium; ^8^ KU Leuven, Department of Neurosciences Research Group Experimental Neurosurgery and Neuroanatomy Leuven Belgium; ^9^ KU Leuven, Laboratory for Cognitive Neurology Department of Neurosciences Leuven Belgium

**Keywords:** constrained spherical deconvolution, diffusion tensor imaging, direct electrical stimulation, neurosurgery, presurgical planning, white matter

## Abstract

**Objectives:**

Accurate presurgical brain mapping enables preoperative risk assessment and intraoperative guidance. This cross‐sectional study investigated whether constrained spherical deconvolution (CSD) methods were more accurate than diffusion tensor imaging (DTI)‐based methods for presurgical white matter mapping using intraoperative direct electrical stimulation (DES) as the ground truth.

**Methods:**

Five different tractography methods were compared (three DTI‐based and two CSD‐based) in 22 preoperative neurosurgical patients undergoing surgery with DES mapping. The corticospinal tract (CST, *N* = 20) and arcuate fasciculus (AF, *N* = 7) bundles were reconstructed, then minimum distances between tractograms and DES coordinates were compared between tractography methods. Receiver‐operating characteristic (ROC) curves were used for both bundles. For the CST, binary agreement, linear modeling, and posthoc testing were used to compare tractography methods while correcting for relative lesion and bundle volumes.

**Results:**

Distance measures between 154 positive (functional response, pDES) and negative (no response, nDES) coordinates, and 134 tractograms resulted in 860 data points. Higher agreement was found between pDES coordinates and CSD‐based compared to DTI‐based tractograms. ROC curves showed overall higher sensitivity at shorter distance cutoffs for CSD (8.5 mm) compared to DTI (14.5 mm). CSD‐based CST tractograms showed significantly higher agreement with pDES, which was confirmed by linear modeling and posthoc tests (*P*_FWE_ < .05).

**Conclusions:**

CSD‐based CST tractograms were more accurate than DTI‐based ones when validated using DES‐based assessment of motor and sensory function. This demonstrates the potential benefits of structural mapping using CSD in clinical practice.

## Graphical abstract

Presurgical white matter mapping using probabilistic CSD tractography is more accurate and sensitive than manual DTI FACT or automated probabilistic DTI tractography. This study included 22 patients with DES data, which was used as the ground truth. Distance in mm between tractograms and DES data resulted in 860 datapoints, 685 of which belonged to the CST and were used for linear modeling; AUC, area under the curve; CSD, constrained spherical deconvolution; DTI, diffusion tensor imaging; FWE, family‐wise error rate; TCK, tractogram/tractography.

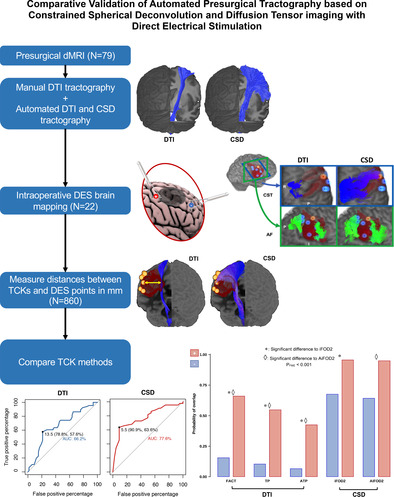

**List of abbreviations**
A: anterior
ACT: anatomically constrained tractography
AF: arcuate fasciculus
AiFOD2: anatomically constrained second order integration over fiber orientation distributions
ANTs: advanced normalization tools
AP: antero‐posterior
ATP: anatomically constrained tensor probabilistic
BIDS: brain imaging data structure
BW: bandwidth
CSD: constrained spherical deconvolution
CST: corticospinal tract
DES: direct electrical stimulation
DF: degrees of freedom
dMRI: diffusion magnetic resonance imaging
DSC: dice similarity coefficient
DTI: diffusion tensor imaging
EPI: echo‐planar imaging
FA: flip angle
FACT: fiber assignment by continuous tracking
FLAIR: fluid attenuation inversion recovery
fMRI: functional magnetic resonance imaging
FSL: functional magnetic resonance software library
FT: fiber tractography
FWE: family‐wise error
HARDI: high nngular resolution diffusion imaging
iFOD2: second order integration over fiber orientation distributions
IQR: interquartile range
ITK: insight toolkit
JI: Jaccard index
KUL_FWT: KU Leuven fun with tracts
KUL_NIS: KU Leuven neuroImaging suite
mm: millimeters
MRI: magnetic resonance imaging
ms: milliseconds
nDES: negative direct electrical stimulation
NPV: negative predictive value
P: posterior
*p*_FWE_
: family wise error rate corrected *p*‐values
*p*_uncorr_
: uncorrected *p*‐values
PA: postero‐anterior
pDES: positive direct electrical stimulation
PE: phase encoding
PPV: positive predictive value
PT: patient
ROC: receiver operating characteristic
ROIs: regions of interest
RQ: research question
s/mm^2^
: seconds per squared millimeter
SENSE: sensitivity encoding in plane parallel imaging acceleration
synB0‐DisCo: synthetic‐B0 distortion correction
T: T‐statistic
TE: echo time
TIV: total intracranial volume
TP: tensor probabilistic
TR: repetition time
VBG: virtual brain grafting

**Practitioner Points**

- Presurgical probabilistic tractography based on CSD is more sensitive than probabilistic and deterministic DTI‐based tractography.
- CSD‐based tractography produces more anatomically complete representations of white matter bundles compared to DTI.
- Presurgical tractography can be fully automated with comparable accuracy to manual methods.

## 1 INTRODUCTION

Modern neurosurgery strives to optimize functional preservation and therapeutic outcomes. The gold‐standard for function‐preserving brain surgery (Gogos et al., 2020) is intraoperative direct electrical stimulation (DES) during awake‐surgery with image‐guided neuronavigation. Within this framework, DES serves a dual purpose: it helps in mapping baseline brain function prior to resection and assists in ascertaining the safety of excision margins during the resection of brain tumors and epileptic foci, for example. Magnetic resonance imaging (MRI)‐based non‐invasive brain mapping, normally complements and guides DES (Dadario et al., 2021; Gharabaghi & Tatagiba, 2007; Schebesch et al., 2019; Wengenroth et al., 2011). However, it may be the sole mapping method available in patients where the gold‐standard is not feasible due to its complexity and potential complications (Burnand & Sebastian, 2014; Dreier et al., 2009; Jones & Smith, 2004).

In this cross‐sectional study we focused on presurgical mapping with diffusion MRI (dMRI)‐based fiber tractography (FT). FT is the rendering of white matter fasciculi in 3D using dMRI signal contrast and computational modeling (Catani et al., 2002). Due to its clinical accessibility and validation (Bello et al., 2011; Coenen et al., 2016; Javadi et al., 2017; Küpper et al., 2015; Nowacki et al., 2018), the most commonly used FT approach in neurosurgical settings is diffusion tensor imaging (DTI)‐based fiber assignment by continuous tracking (FACT) (Mori et al., 1999).

DTI‐FACT suffers from multiple limitations making it suboptimal in neurosurgical practice. DTI assumes a single fiber direction per voxel, which compromises the accuracy of modeled streamline trajectories in voxels containing complex fiber architecture, leading to underestimation of the true extent of fiber bundles (Farquharson et al., 2013; Mormina et al., 2015). DTI‐FACT is also confounded by lesion‐effects, as perilesional edema can compromise sensitivity to fiber orientation due to reduction in fractional anisotropy and increase in mean diffusivity, resulting in false‐negatives, that is, missing streamlines/tracts. Additionally, FT suffers from user‐bias and limited reproducibility when manual region of interest (ROIs) definition is used, which is the most common approach in clinical practice. These short‐comings limit the accuracy of presurgical fiber tracking and motivate exploring alternative methods using automation, and higher‐order model based tractography (Farquharson et al., 2013).

High angular‐resolution imaging (HARDI) methods like constrained spherical deconvolution (CSD) (Tournier et al., 2007) can address the limitations of DTI‐FACT (Anderson, 2005; Tournier et al., 2004; Tuch et al., 2002, 2003; Wedeen et al., 2000, 2005, 2008). However, HARDI typically requires a more complex data acquisition compared to DTI (Calamante & Connelly, 2009; Dell'Acqua & Tournier, 2019; Jeurissen et al., 2014; Tournier et al., 2013). Some techniques, such as CSD have been shown to improve tractography based on single‐shell low angular‐resolution clinical data (Calamuneri et al., 2018; Toselli et al., 2017). Probabilistic‐FT tends to have a higher sensitivity compared to deterministic‐FT, and thus may be preferable in a surgical setting where false‐negatives are more critical. Anatomically‐constrained tractography (ACT) (Smith et al., 2012) may also improve accuracy of clinical tractography by constraining streamline origin/termination to the gray‐white matter interface.

In this study we compared distance measures between DES coordinates, acquired intraoperatively through pathology‐tailored craniotomies, and tractograms generated with deterministic DTI‐FACT, probabilistic‐DTI, and probabilistic‐CSD, with and without ACT, to determine their respective suitability for presurgical mapping. We focused on the corticospinal tract (CST) and arcuate fasciculus (AF) given their relevance in clinical practice as primary pathways for sensory‐motor and language functions. This approach is based on the premise that DES coordinates that resulted in an observable response were indicative of structural connectivity to white matter tracts of interest, while DES coordinates that resulted in no observable response were indicative of a lack of structural connectivity to the tracts of interest (Coenen et al., 2016; Essayed et al., 2017).

## 2 MATERIALS AND METHODS

### 2.1 Study design and research questions

We conducted an observational study among 22 surgery‐naïve patients to compare the performance of CSD‐ and DTI‐based tractography with DES as the ground truth. The methodology involved multimodal presurgical MRI scanning and intraoperative brain mapping. Our research aimed to address the following questions:

**RQ1:** How do distance measures between tractograms and DES coordinates compare among different tractography methods?

**RQ2:** How do these methods compare in terms of binary agreement/disagreement with DES at various distance cutoffs?

**RQ3:** Are differences in tractography methods for the corticospinal tract (CST) driven by bundle volume and/or lesion volume, or is there a genuine difference in accuracy between CSD and DTI tractograms when compared to DES?

To address these questions, we utilized distance measures, ROC curves, and a two‐part linear mixed model.

### 2.2 Participants

Patients were informed about the study and signed a written informed consent before participation, in accordance with the declaration of Helsinki. Local ethics committee approval was obtained (UZ/KU Leuven, Leuven, Belgium, S61759). Participating patients were excluded if they had undergone previous resective brain surgery, had brain implants, ventriculoperitoneal shunts, or had absolute contraindications to MRI. We recruited 79 surgery‐naïve patients referred for presurgical fMRI and DTI between 01/2019 and 01/2021, 22 patients also underwent intraoperative DES mapping (14 males, age = 8–73 years, median = 39.5, IQR = 28, neoplasms = 18, and focal cortical dysplasia = 4). Summarized demographics and pathological information can be found in Table 1 and further detailed in Table S1.

**TABLE 1:** Summarized demographics, pathological type, distribution, and volumes.

Age and gender	Lesion			
	Type	Side	Cerebral lobar distribution	Lesion volumes
Age range = 8–73 years Median age = 39.5 years IQR = 28 years 14 males 8 females	18 neoplasms: 16 gliomas (10 HGG) 1 meningioma 1 dysembryoplastic neuro‐epithelial tumor 4 focal cortical dysplasia	10 R 12 L	3 fronto‐parietal 1 fronto‐temporal 11 frontal 3 parietal 1 parieto‐occipital 2 temporo‐fronto‐parietal 1 fronto‐temporo‐parieto‐occipital (multifocal lesion)	Range = 1.2–232.13 mL Median = 44.70 IQR = 71.74

Abbreviations: F, female; HGG, high grade glioma; IQR, interquartile range; L, left; M, male; mL, milliliters; PT, patient; R, right.

### 2.3 MRI acquisition

Two 3‐Tesla MRI scanners were used for multimodal presurgical MRI scanning (Ingenia – Elition, and Achieva dStream, Philips Medical Systems, Best, The Netherlands), both with 32‐channel receive head coils. The acquisition parameters for 3D T1‐weighted images, T2‐ and T2 fluid attenuation inversion recovery (FLAIR) images were previously described (Radwan et al., 2021). Multi‐shell dMRI data (*b*‐value 1200 and 2500 s/mm^2^) and reversed phase b0 images were acquired whenever feasible and tolerated by the patient. Single‐shell data with or without reversed phase B0 images were used if multi‐shell data could not be acquired, for further details see Tables S2 and S3.

### 2.4 Intraoperative brain mapping

Twenty patients underwent awake neurosurgery and DES, and two patients underwent DES with motor and somatosensory evoked potentials (MEP/SSEP). Intraoperative frameless neuronavigation (Curve, BrainLab, Munich, Germany) was employed in all cases. DES used the OSIRIS neurostimulator (Inomed Medizintechnik GmbH, Germany), and a bipolar fork stimulator with 5 mm inter‐electrode spacing for cortical mapping, and a monopolar suction‐stimulator for subcortical mapping.

DES stimulation parameters followed the protocol described by Duffau et al. (1999) (60 Hz) in anesthetized patients, and the low frequency protocol described by Zangaladze et al. (2008) (5 Hz) in awake patients. Stimulation started with 2–4 mA and gradually increased to 20 mA, or until a positive response was found. DES coordinates were considered positive (pDES) if stimulation interfered with task performance or elicited a motor or sensory effect reported by the patient, observed by the attending neurophysiologist, or recorded on MEP/SSEP. DES coordinates were considered negative (nDES) if no response could be elicited up to 20 mA in stimulation amplitude, and no effects were found if resection approached its location.

Baseline cortical DES mapping was done before resection and immediately after opening the dura and waking the patient, in case of awake surgery. Testing locations were chosen based on visible anatomical landmarks in the surgical field and the co‐registered MR images. DES tested locations were marked by sterile square markers, registered in the neuronavigation system, and repeatedly tested during resection. Cortical and subcortical coordinates were saved and included in this analysis. The choice of DES mapping approach was based on lesion location and awake surgery feasibility. The bundles of interest were defined based on the neurosurgical treatment plan. Table S3 lists the details of the DES protocol per patient. To account for potential brain‐shift effects, screenshots were acquired on the neuronavigator when saving cortical and subcortical DES coordinates to ensure concordance between the images and the marked locations.

### 2.5 Data analysis

**FIGURE 1:**
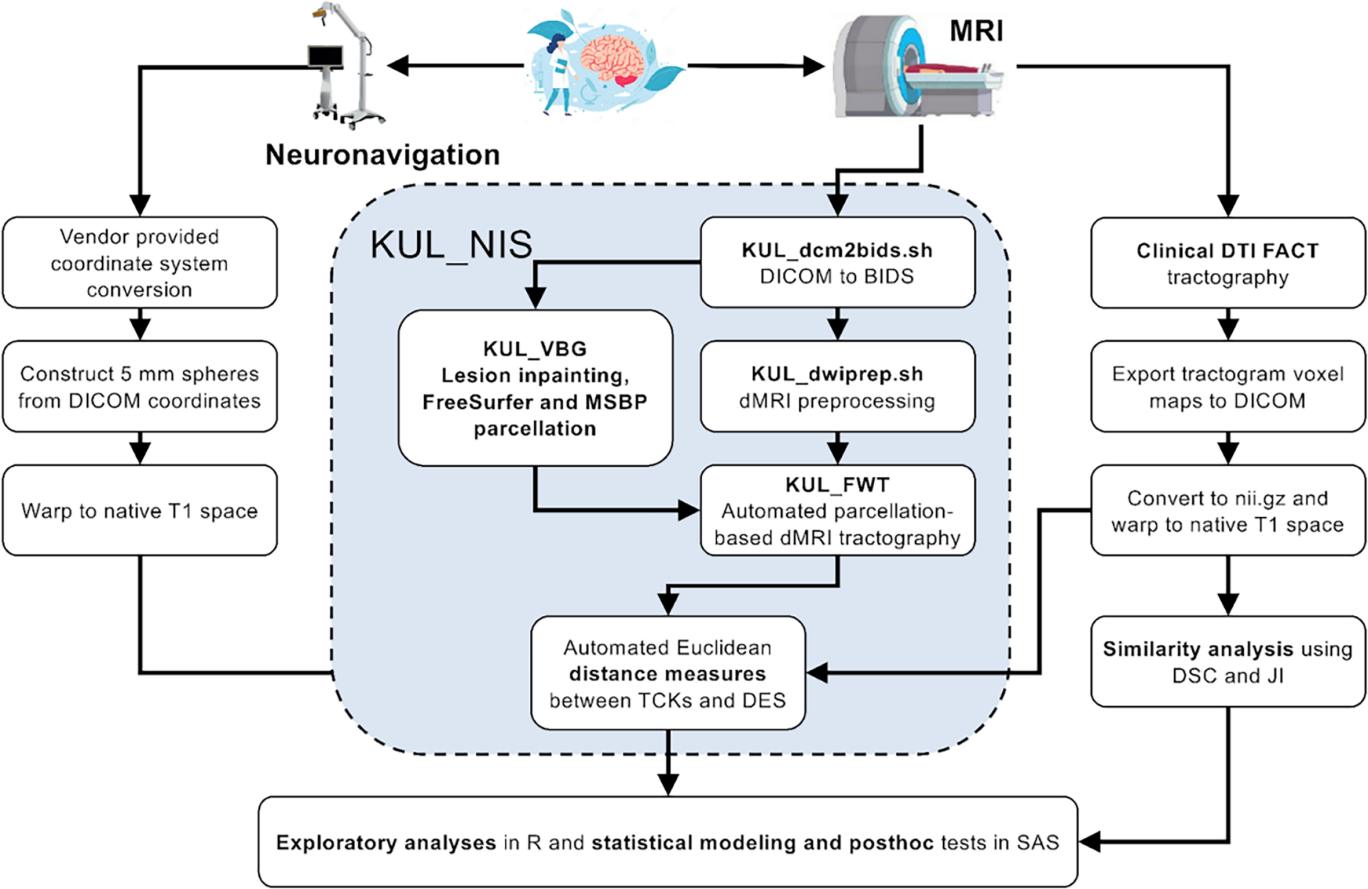
Schematic representation of the data preprocessing and analysis workflow used to compare different tractography results to intraoperative mapping outcome. BIDS, brain imaging data structure; DSC, dice similarity coefficient; DTI, diffusion tensor imaging; FACT, fiber assignment by continuous tracking; JI, Jaccard index; KUL_FWT, KU Leuven fun with tracts; KUL_NIS, KU Leuven neuroimaging suite; KUL_VBG, KU Leuven virtual brain grafting; MRI, magnetic resonance imaging.

Figure 1 shows a schematic representation of the data preprocessing and analysis workflow. All acquired images were converted to the brain imaging data structure (BIDS) (Gorgolewski et al., 2016) format using the KU Leuven Neuroimaging suite (KUL_NIS) (*KULeuven Neuro Imaging Suite (KUL_NIS)*, 2018/2022) (https://github.com/treanus/KUL_NIS) and dcm2bids (Bedetti et al., 2022).

#### 2.5.1 Structural images

Lesion segmentation was done by a single‐rater (AR, neuroradiologist with 10 years of experience) based on the T1, T2, T2‐FLAIR, and contrast‐enhanced T1‐weighted images using the semi‐automated classification approach in ITK‐snap v3.8.0 (Yushkevich et al., 2019). Manual segmentation using the brush or smart brush tools was used in case of failure of the semi‐automated approach. KU Leuven Virtual brain grafting v0.52 (VBG) (Radwan et al., 2021) (https://github.com/KUL-Radneuron/KUL_VBG) was used for T1 lesion‐filling and T1 parcellation (Fischl, 2012; Henschel et al., 2020; Tourbier et al., 2019). FreeSurfer (Fischl, 2012) generated 1 mm isotropic T1‐weighted images were used as base images for calculating distances. Lesion volume, total intracranial volume (TIV), and lesion mask to TIV ratios were also calculated per patient.

#### 2.5.2 Diffusion‐weighted images

Routine clinical dMRI analysis involved in‐line rigid interframe registration for motion correction on a clinical workstation (Philips Medical Systems, Best, The Netherlands). Advanced dMRI preprocessing used the KUL_dwiprep.sh script (*KULeuven Neuro Imaging Suite (KUL_NIS)*, 2018/2022), which relies on FSL v6.0 (Jenkinson et al., 2012), ANTs v2.3.0 (Avants et al., 2011; Tustison et al., 2021) and MRtrix3 v3.0.3 (Tournier et al., 2019) for denoising, correction of Gibbs ringing, subject motion, Eddy current artifacts, echo‐planar imaging (EPI) distortion, and imaging bias. FSL's topup (Andersson et al., 2003) was used for EPI distortion correction if reversed phase B0 images were available, and synB0‐DisCo v3.0 (Schilling et al., 2019) if not. This was followed by model fitting for DTI, using dwi2tensor, and CSD, using dwi2response and dwi2fod in MRtrix3 (Dhollander et al., 2016, 2018; Tournier et al., 2019). Multi‐shell multi‐tissue CSD (Jeurissen et al., 2014) was used to estimate CSD for two tissues (WM and CSF + GM) for single and multi‐shell data.

#### 2.5.3 Tractography

Five different tractography methods were included. The standard clinical DTI‐FACT based on manual white matter ROIs delineated (Catani & Thiebaut de Schotten, 2008) by two experienced operators on the Philips FiberTrack software was used as the reference method (FACT). Additionally, we included four automated approaches using probabilistic tensor‐based tractography (TP) (Jones, 2008), and anatomically‐constrained TP (ATP), and probabilistic‐CSD tractography using second order integration over fiber orientation distributions (iFOD2) (Tournier et al., 2010), and anatomically‐constrained iFOD2 (AiFOD2). This resulted in five different versions of every bundle of interest.

Automated tractography (TP, ATP, iFOD2, and AiFOD2) was done using the bundle‐specific approach in KU Leuven Fun With Tracts v0.6 (KUL_FWT) (Radwan et al., 2022) (https://github.com/KUL-Radneuron/KUL_FWT) with the addition of a model‐based tractogram filtering step using streamline clustering (see Appendix S1). Tckgen's (Tournier et al., 2019) default settings were used for tractography except for maximum angle threshold, which was set to 45°, minimum and maximum fiber lengths were set to 50 and 280 mm, and 15,000 (CST) and 6000 (AF) streamlines were required for the initial bundles. The number of streamlines required initially were empirically determined based on the minimum number required to generate the most anatomically representative visualization of the tract in every dataset, for example, fanning of the CST into lateral and inferior primary sensory‐motor cortex, extension of AF into inferior frontal and superior temporal gyri. Further details are described in the supplementary data of Radwan et al. (2022). All generated tractograms were visually assessed for quality before further analysis. ANTs (Avants et al., 2011; Tustison et al., 2021) registration was used to bring all results to each subject's T1 space for quantitative comparisons. Voxel‐wise volumes for each bundle and bundle to TIV ratios were calculated per patient.

#### 2.5.4 DES coordinates processing and distance measures

Saved DES coordinates were exported from the neuronavigator in the proprietary format Xbrain and converted to mm using a proprietary BrainLab script “Xbrain to points”. Spheres with 5 mm radii (Cochereau et al., 2016) were created around each DES coordinate with FSL (Jenkinson et al., 2012) in the same space as the anatomical image used during surgery, then warped to T1 space with ANTs (Avants et al., 2011; Tustison et al., 2021) for comparison with the tractography results. Minimum Euclidean distances were calculated between all DES coordinates and all voxels (cortical and subcortical) of the corresponding tractogram in Python v3.8. The DES spheres in each patient's native T1 space were collapsed to their centers of mass (COM) and recreated with the same radii to mitigate deformations resulting from registration.

The following Python packages were used in an automated script in Python v3.8 to measure the minimum Euclidean distances between DES coordinates and tractograms: nibabel v3.2.2 (Brett et al., 2022), numpy v1.22.3 (Harris et al., 2020), scipy v1.4.1 (Virtanen et al., 2020). Distance measures were rounded up to integers (in mm) because increments smaller than a single voxel (1 mm) were not considered meaningful.

### 2.6 Statistical testing

#### 2.6.1 Exploratory analysis

Distances between DES coordinates and tractograms can be considered akin to screening test results, and can thus be compared using confusion matrices, and ROC curves. However, due to the small sample size, and intersubject variation in number of DES coordinates, tractograms, and lesion size, we opted for a more in‐depth analysis. First (RQ1) we plotted the raw distance measures (Patil, 2021), then distances were averaged for each patient per DES response type to remedy the bias for patients with higher number of datapoints and ROC (Robin et al., 2011) curves were used to explore differences in sensitivity, specificity, and Youden's index (Youden, 1950) ideal cutoffs.

Binary agreement/disagreement rates for Research Question 2 (RQ2) were determined at varying distance thresholds using confusion matrices. In this context, pDES indicated the presence of eloquent brain tissue, while a lack of response signified its absence. Tractogram‐DES pairs with distances below the cutoff were considered positive datapoints: true‐positives for pDES and false‐positives for nDES. Conversely, pairs exceeding the cutoff were negative datapoints: true‐negatives for nDES and false‐negatives for pDES.

#### 2.6.2 Two‐part linear modeling

To further investigate the difference in performance between tractography methods while accounting for the intersubject nesting, the excess zeros, tractogram volumes, lesion volumes, and brain volume (RQ3). Further, to account for potential uncertainty of DES coordinate precision due to subject‐specific phenomenon (e.g., brain shift), we applied a minimum threshold based on the ROC determined cutoff from all pooled averaged data. Resulting thresholded distances data were a semicontinuous variable (integers in mm) with excess zeros and an extremely right‐skewed distribution, violating assumptions of normality. Therefore, and given the within‐subjects nesting of repeated distance measures, we opted for a two‐part model for longitudinal data (Farewell et al., 2017; Tooze et al., 2002). The model was estimated with the %MIXCORR macro provided by Tooze et al. (2002) and PROC NLMIXED in SAS studio v9.4 (SAS Institute, Cary, NC, USA).

Spatial overlap between tractograms and pDES were expected to be more common compared to nDES coordinates, while nDES coordinates were expected to be generally more distant from tractograms. The first part (A) predicted the probability of overlap (distance = 0), and the second part (B) predicted the distances between nonoverlapping (distance > 0) tractogram‐DES coordinate pairs. Distances were the dependent variable and DES response, and tractography method (FACT, TP, ATP, iFOD2, and AiFOD2) were used as predictors in both parts of the model. Bundle‐to‐TIV and lesion‐to‐TIV ratios, were used as covariates of noninterest. Adaptive Hochberg's (Hochberg & Benjamini, 1990) family‐wise error‐rate (FWE) correction was used to control for type(I) error in posthoc testing. Further details can be found in Appendix S1.

## 3 RESULTS

### 3.1 Lesion segmentation, inpainting and tractography

**FIGURE 2:**
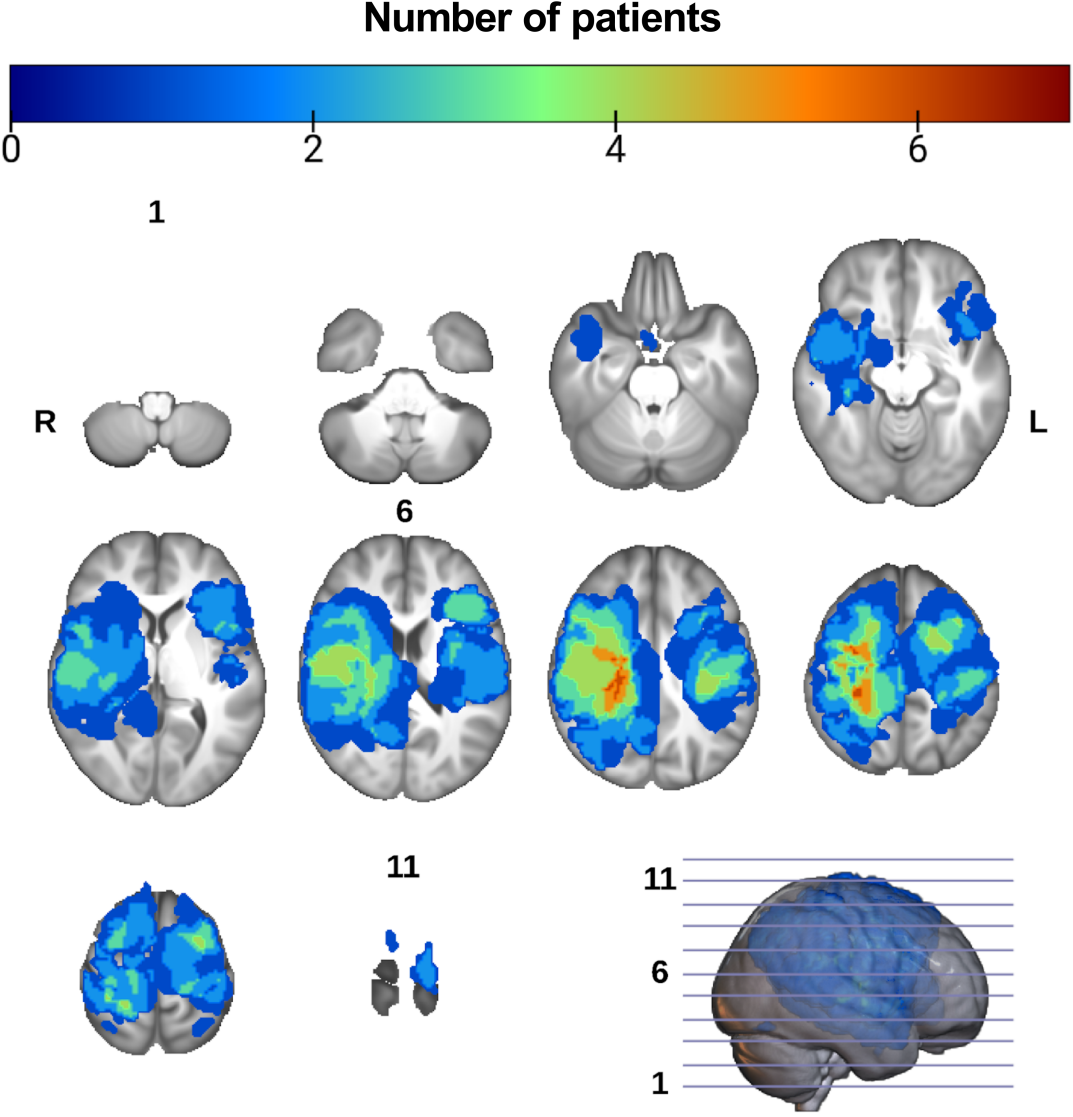
Spatial distribution of lesions from all patients overlaid onto the UK biobank T1 template brain in standard Montreal neurological institute (MNI) space. Perilesional edema was included in the masks of neoplasms if present. Overlay voxel intensities correspond to the sum of overlapping lesion masks from different patients. L, left; R, right; slice numbers are indicated for the first, middle, and last slices.

**FIGURE 3:**
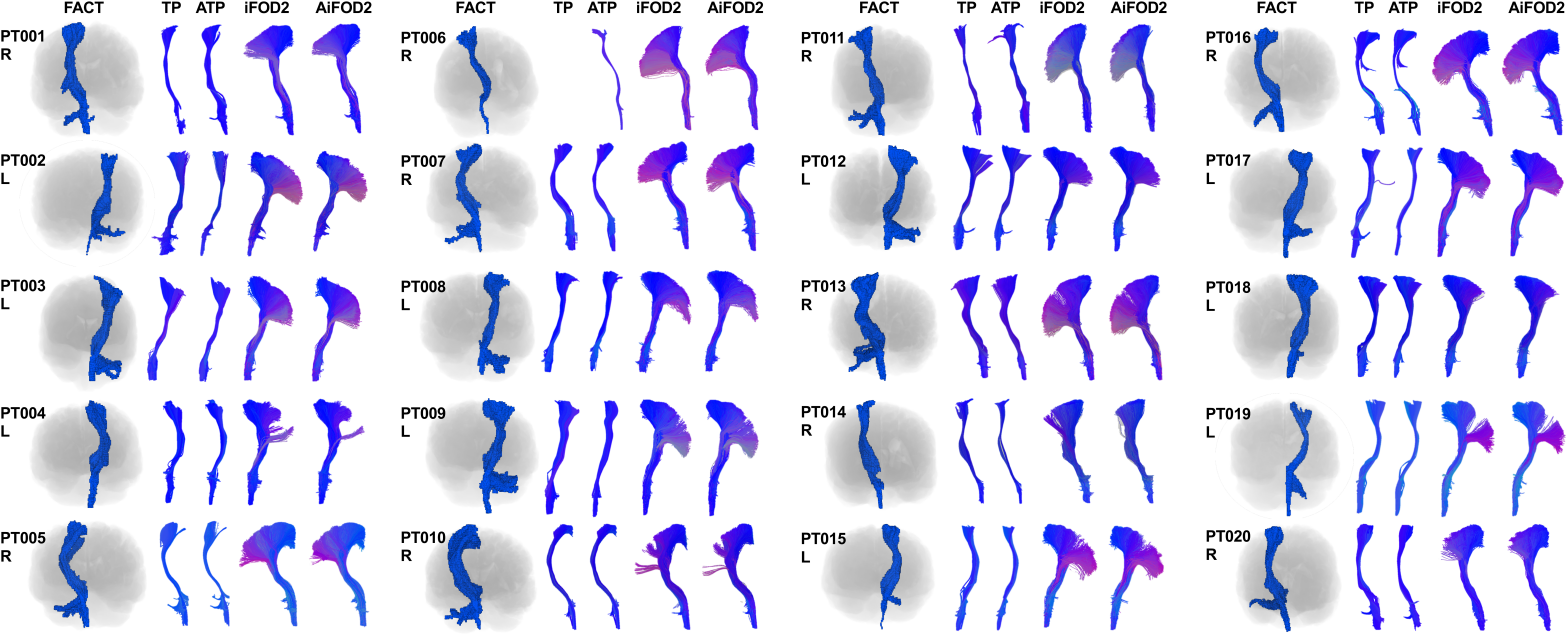
Corticospinal tractograms representative images from all methods in anterior view. The FACT tractogram outputs shown in blue are generated from volume rendered voxel masks with the T1‐brain image silhouette shown underneath. The other four methods TP, ATP, iFOD2, and AiFOD2 are shown as 3D rendered streamlines with end‐point directional color coding. All images are shown in radiological orientation. AiFOD2, anatomically constrained iFOD2; ATP, anatomically constrained tensor probabilistic; FACT, fiber assignment by continuous tracking; iFOD2, probabilistic tractography by second order integration over spherical harmonics; PT, patient; TP tensor probabilistic.

Lesion masks had a median volume = 44.70 mL, minimum = 1.20, maximum = 232.129, and IQR = 67.74 mL. Figure 2 shows the cumulative voxel‐wise distribution of lesions in this sample of patients over the whole brain in MNI152 space. Lesion‐inpainting and structural parcellation was successful in all patients. Tractography yielded 134 out of 135 attempted bundle reconstructions, with one failed right CST TP tractography (PT006). No bundles were excluded on inspection, and none were repeated. Figures 3 and S1 show all generated CST and AF tractograms, respectively. All methods showed a good level of visual agreement, but CSD‐based methods resulted in more extensive tractograms. CSD captured the characteristic fanning of the CST into the lateral and inferior sensory‐motor cortex, while DTI did not show this appearance, and AF CSD reconstructions reached further into the inferior frontal gyri and temporal lobes compared to DTI. As part of tractogram quality analysis we also explored the similarity of bundles generated with different FT methods, see Appendix S1 and Table S4 for details.

### 3.2 Intraoperative mapping and distance measures

**FIGURE 4:**
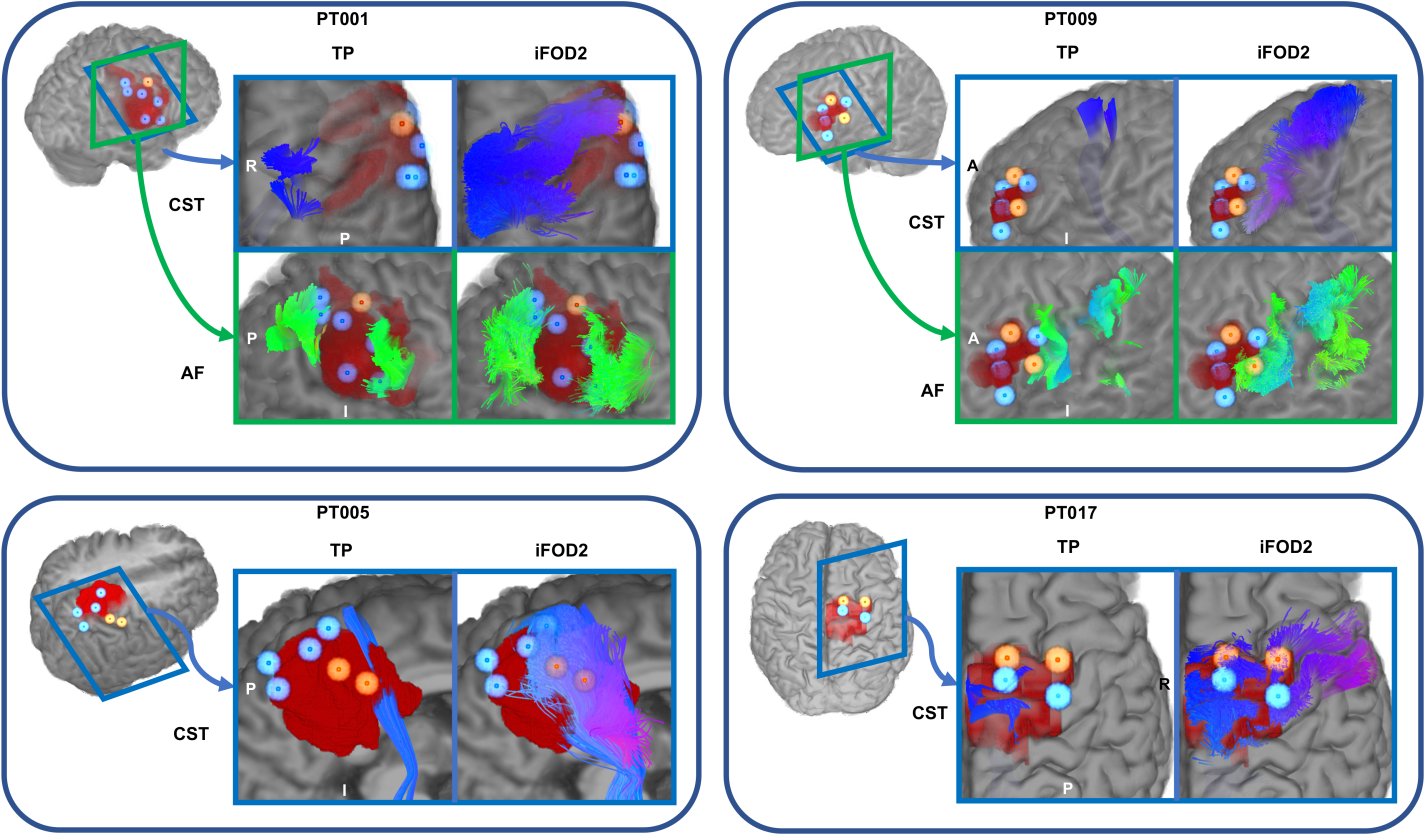
DES spheres, lesion masks, and tractograms overlaid onto surface‐rendered T1 images, for four example patients as generated with TP and iFOD2. Positive DES spheres are shown in semitransparent orange and their centers are shown in opaque orange, negative DES spheres are shown in semitransparent light blue with opaque blue centers. Tractograms are shown in endpoint‐based directional color coding. A, anterior; AF, arcuate fasciculus; CST, corticospinal tract; I, inferior; iFOD2, probabilistic tractography based on second order integration of spherical harmonic distributions; P, posterior; R, right; TP, tensor probabilistic.

**FIGURE 5:**
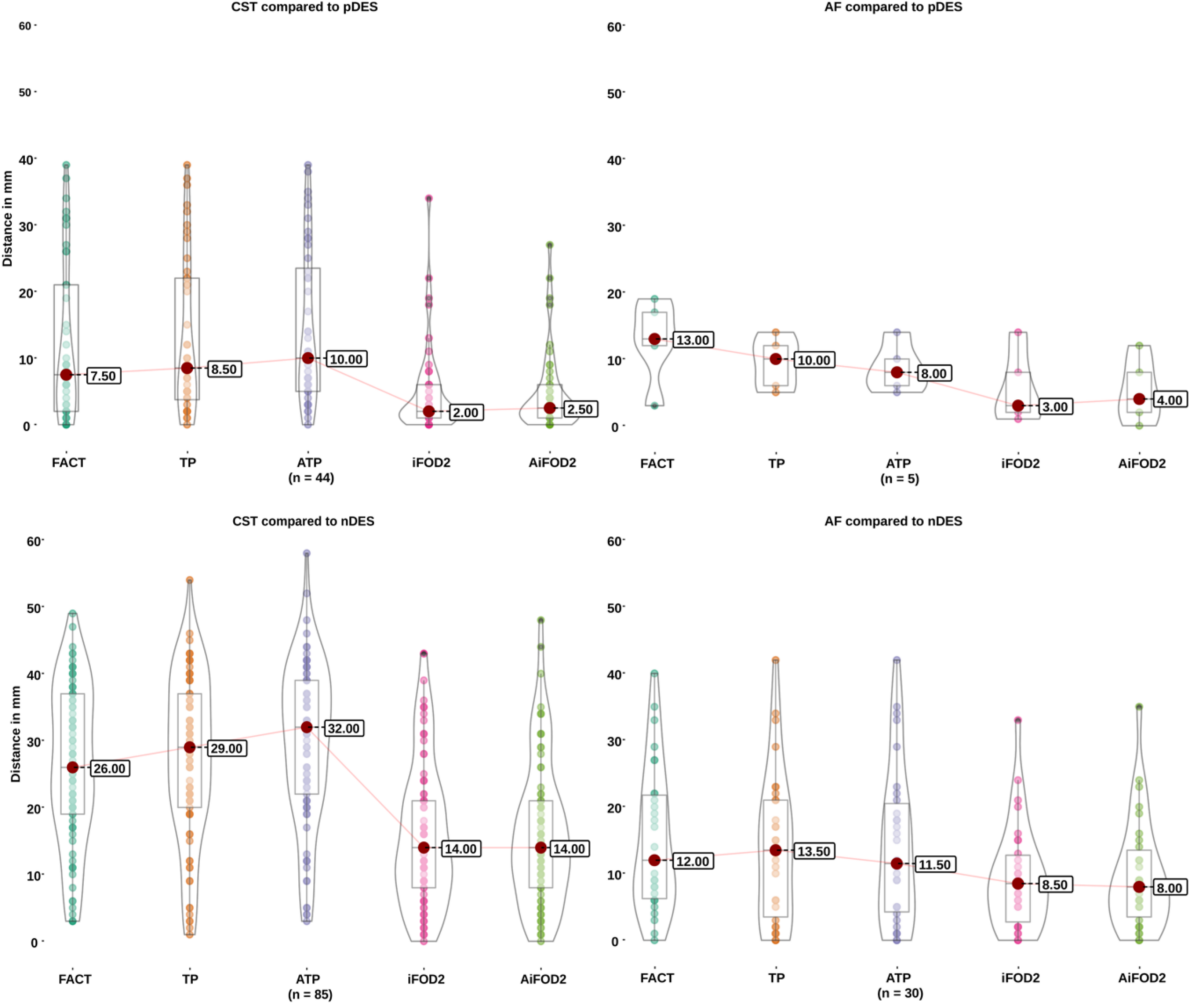
Box and violin plots of distance measures by bundle type, DES response type and tractography method. Results for the CST are shown on the left, for the AF on the right, for pDES on top, and for nDES on the bottom. The highlighted circle at the center of each violin plot indicates the median distance value, the box within each violin indicates the interquartile range, and the central line represents the range. ATP, anatomically constrained tensor probabilistic; AiFOD2, anatomically constrained iFOD2; FACT, fiber assignment by continuous tracking; iFOD2, probabilistic tractography by second order integration over spherical harmonics; TP, tensor probabilistic.

DES mapping resulted in 51 pDES and 123 nDES coordinates, 157 cortical (90%) and 17 subcortical (10%), three patients had only nDES coordinates, namely PT011, PT012, and PT022, while three patients had only pDES coordinates, namely PT010, PT013, and PT015. Table S3 lists the DES tests, number of resulting pDES and nDES coordinates, elicited pDES response, acquired dMRI data, and bundle(s)‐of‐interest per patient. Figure 4 shows images from four exemplar cases demonstrating the DES spheres and tractography results. Each tractogram was paired with pDES coordinates, and all nDES coordinates for distance measures, resulting in 860 distance measures with 174 measures for all methods except TP, which had 164 due to the failed right CST for PT006. Table S5 lists summarized descriptive statistics for distance measures, and Figure 5 shows the distribution of distances per bundle and DES response.

### 3.3 Statistical testing

#### 3.3.1 Exploratory analysis

**FIGURE 6:**
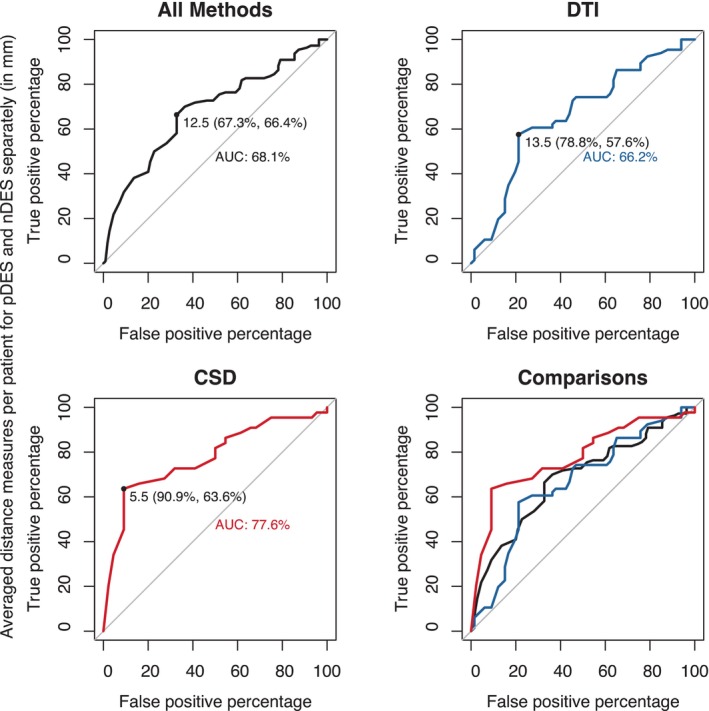
ROC curves and optimal distance cutoffs calculated using Youden's method. Results for pooled data from all methods are shown in black, DTI (FACT, TP, and ATP) in blue, and CSD (iFOD2 and AiFOD2) in red; AiFOD2, anatomically constrained iFOD2; ATP, anatomically constrained tensor probabilistic; FACT, fiber assignment by continuous tracking; iFOD2, probabilistic tractography by second order integration over spherical harmonics; TP, tensor probabilistic.

The CST and AF tractograms tended to be closer to pDES than to nDES coordinates, regardless of FT method. Both bundles CSD‐based tractograms showed shorter distances than DTI‐based tractograms to both nDES and pDES coordinates (RQ2), see Figure 5 for plots of unthresholded distances. ROC curves for distance measures of all FT methods pooled together, as well as for both CSD‐based and DTI‐based FT methods are shown in Figure 6 and Figures S2 and S3 show the ROC curves for pooled raw distances, and each FT method separately with and without averaging.

All ROC curves showed notable increase in sensitivity and comparatively smaller decrease in specificity for CSD‐based compared to DTI‐based FT, but there were no significant differences in AUC on pairwise DeLong tests, see Table S6. Youden's index determined distance cutoffs were also notably smaller for CSD‐based methods (pooled = 5.5 mm, iFOD2 = 6.5 mm, and AiFOD2 = 5.5 mm) than DTI‐based methods (pooled = 13.5 mm, FACT = 19.5 mm, TP = 13 mm, and ATP = 14.5 mm), and the cutoff determined from all pooled data was 12.5 mm. CSD‐based methods were also associated with higher sensitivity at all ROC determined thresholds.

We used the three cutoffs determined from ROCs of pooled data of all methods, CSD, and DTI to define binary agreement/disagreement counts (RQ2) to compare FT methods using confusion matrices, see Table 2. These showed higher sensitivity and lower specificity for CSD‐based compared to DTI‐based tractography in both bundles at all distance cutoffs.

**TABLE 2:** Summarized binary agreement/disagreement confusion matrix results for all tractogram‐DES pairs.

FT methods	Thresholds (mm)	Accuracy (%)	Sensitivity (%)	Specificity (%)	PPV (%)	NPV (%)
FACT	5.5	75	33	92	63	77
	12.5	71	61	75	50	82
	13.5	71	65	74	51	83
TP	5.5	68	39	80	45	76
	12.5	70	67	72	49	84
	13.5	70	67	71	49	84
ATP	5.5	69	25	87	45	74
	12.5	72	57	78	52	81
	13.5	72	59	77	52	82
iFOD2	5.5	74	65	77	54	84
	12.5	57	88	45	40	90
	13.5	57	90	43	40	91
AiFOD2	5.5	75	71	77	56	86
	12.5	60	92	47	42	94
	13.5	58	92	44	41	93

Abbreviations: DES, direct electrical stimulation; FT, fiber tractography; NPV, negative predictive value; PPV, positive predictive value.

#### 3.3.2 Two‐part model

The two‐part linear model (RQ3) showed a significant main effect of tractography methods, and DES response type in both parts of the model. CSD‐based methods (iFOD2 and AiFOD2) had larger probabilities of overlap (<12.5 mm) and smaller distances (>12.5 mm) compared to DTI‐based methods (FACT, TP, and ATP) for both pDES and nDES coordinates. Results also showed that pDES coordinates had significantly higher probability of overlap (*T*(18) = −7.70, *p* < .001), and were closer to tractograms than nDES coordinates were (*T*(18) = −5.09, *p* < .001). Bundle‐to‐TIV ratio had a significant effect (*T*(18) = −2.55, *p* = .020) only in the logistic part.

**FIGURE 7:**
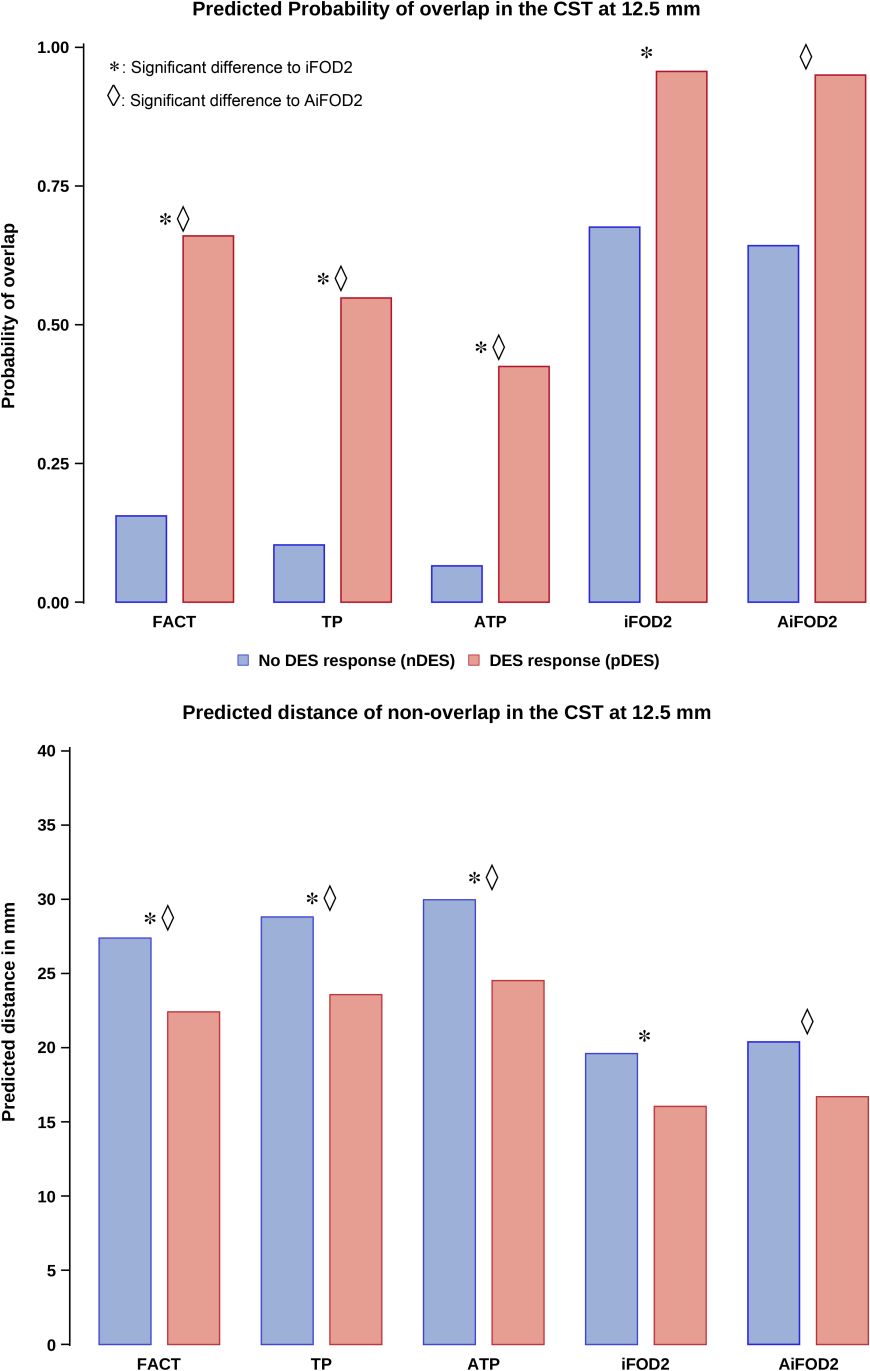
Bar plots for predicted probability of overlap between the CST and DES coordinates (top) and predicted distances to nonoverlapping DES coordinates (bottom) at 12.5 mm distance cutoff. CSD methods showed significantly higher probability of overlap, and lower distance if not overlapping compared to DTI methods. Differences between nDES and pDES were comparable between FT methods. AiFOD2, anatomically constrained iFOD2; ATP, anatomically constrained TP; CST, corticospinal tract; FACT, fiber assignment by continuous tracking; iFOD2, probabilistic tractography by second order integration over spherical harmonics; TP, tensor probabilistic.

No significant interaction was found between DES response type and tractography method for the CST, indicating that differences between nDES and pDES were not significantly different between FT methods. Posthoc testing controlling for lesion‐to‐TIV and bundle‐to‐TIV ratios showed that CSD‐based tractograms were more likely to overlap with and be closer to DES coordinates compared to DTI‐based tractograms. These results are detailed in Table 3 and Figure 7, while Figure S4 and Table S7 show results with 10.5 mm distance cutoff.

**TABLE 3:** Results of post hoc tests after two‐part linear modelling for the CST at 12.5 mm distance cut‐off.

Tractography methods	Model part A				Model part B			
	*T*	CI low/high	*P*_uncorr_	*P*_FWE_	*T*	CI low/high	*P*_uncorr_	*P*_FWE_
FACT versus TP	−1.08	−1.388/0.447	.296	.591	−1.27	−0.134/0.033	.220	.783
FACT versus ATP	−2.19	−1.893/−0.041	.041	.083	−2.36	−0.170/−0.010	.030	.195
FACT versus iFOD2	**5.98**	**1.575/3.282**	**<.001**	**<.001**	**5.51**	**0.158/0.353**	**<.001**	**<.001**
FACT versus AiFOD2	**5.62**	**1.428/3.131**	**<.001**	**<.001**	**6.42**	**0.198/0.391**	**<.001**	**<.001**
TP versus ATP	−1.13	−1.420/0.427	.273	.546	−1.01	−0.121/0.042	.324	.324
TP versus iFOD2	**6.75**	**1.996/3.801**	**<.001**	**<.001**	**6.49**	**0.207/0.405**	**<.001**	**<.001**
TP versus AiFOD2	**6.46**	**1.855/3.644**	**<.001**	**<.001**	**7.43**	**0.248/0.443**	**<.001**	**<.001**
ATP versus iFOD2	**7.59**	**2.456/4.335**	**<.001**	**<.001**	**7.51**	**0.249/0.442**	**<.001**	**<.001**
ATP versus AiFOD2	**7.32**	**2.315/4.178**	**<.001**	**<.001**	**8.49**	**0.290/0.480**	**<.001**	**<.001**
IFOD2 versus AiFOD2	−0.44	−0.862/0.564	.665	.665	−0.27	−0.069/0.148	.451	.451

*Note*: Bold values denote significant differences on *p*_FWE_.

Abbreviations: AiFOD2, anatomically constrained iFOD2; ATP, anatomically constrained tensor probabilistic; CI, confidence interval; CST, corticospinal tract; Df, degrees of freedom; FACT, fiber assignment by continuous tracking; iFOD2, second order integration over fiber orientation distributions; *P*_uncorr_, uncorrected *p* values; *P*_FWE_, Hochberg family‐wise error rate corrected *p* value; *T*, *t*‐statistic; TP, tensor probabilistic.

## 4 DISCUSSION

The primary aim of this work was to compare the accuracy of CSD and DTI tractography results as evaluated against intraoperative DES stimulation. Our analysis utilized data from a group of 22 preoperative neurosurgical patients for whom DES was being performed during lesion resection (tumors mostly) to determine safe resection margins with respect to eloquent tissue. First, raw distance measures from CSD‐based tractograms to DES coordinates were smaller than for DTI tractograms. These also showed that pDES coordinates were generally closer to tractograms compared to nDES coordinates. ROC curve plots and Youden's index determined distance cutoffs, accuracy, sensitivity, and specificity (RQ1) showed that pooled CSD methods (5.5 mm) had a shorter distance cutoff compared to pooled DTI methods (13.5 mm). This finding held with or without pooling and/or averaging.

Overall higher sensitivity for CSD, and higher specificity for DTI tractograms was also found when comparing FT methods for their binary agreement/disagreement with DES at different distance cutoffs (RQ2) (5.5, 12.5, and 13.5 mm). The cutoff (12.5 mm) determined by Youden's index on the averaged all pooled data ROC was used to define binary agreement/disagreement (≤12.5/>12.5 mm) rates between tractograms and DES.

Lastly, we relied on a two‐part linear mixed model and posthoc testing to investigate the effects of DES response, and differences between FT methods in probability of overlap (agreement) versus nonoverlap (disagreement), and distance measures for nonoverlapping tractogram‐DES pairs while controlling for effects of variation in lesion and bundle volumes (RQ3).

This showed that regardless of FT method, pDES coordinates were significantly closer and more probable to overlap with the CST compared to nDES coordinates. In other words, brain locations giving positive sensory‐motor functional responses on stimulation (pDES), so called eloquent areas, were more likely to overlap with the CST, and if not overlapping they would typically be closer than coordinates giving no functional effect on DES, so called non‐eloquent. Furthermore, CST tractograms generated with CSD were significantly more probable to overlap with and be closer to DES coordinates compared to DTI, regardless of DES response. Posthoc testing confirmed that iFOD2 and AiFOD2 significantly outperformed FACT, TP, and ATP in both parts of the model. These findings also indicate that the differences are not solely driven by larger volumes of CSD tractograms.

DTI‐based tractography, when compared to DES, has previously been reported to have a generally satisfactory performance (Bello et al., 2011; Coenen et al., 2016; Manan et al., 2022). However, a closer look at the literature shows that these studies used manual tractography (Xiao et al., 2021), and/or excluded negative DES coordinates from their analyses (Berman et al., 2007). Additionally, most of the previous work utilized comparatively simple statistical methods and tended not to include covariates such as bundle volume, TIV or lesion volume in their analysis. In contrast to the literature, we found a less than satisfactory performance for DTI‐based FT.

Thus far few studies have compared different tractography methods to DES and/or DBS results (Bucci et al., 2013; Gröschel et al., 2011; Mandelli et al., 2014; Sheng et al., 2021) all of which found a clear benefit from using more advanced tractography methods such as CSD. Our study adds to this growing body of evidence showing that CSD FT improved the representation of functional white matter anatomy based, and that this automated approach performed better than the manual standard of practice with DTI. While this indicates that automated methods relying on accurate structural parcellation adapted to effects of pathology can be comparable to expert users for manual FT. This study did not include manual CSD FT however, which could have performed even better than the automated approach and remains to be investigated in future work.

It must be also noted that all DTI FT methods performed worse than CSD FT as far as distances and agreement with DES coordinates were concerned, while most of the DES coordinates were cortical thus potentially placing DTI at a disadvantage, cortical penetrance however was not qualitatively identified as deficient for tensor‐based FT. Conversely, it is obvious upon inspection of the resulting tractograms that the tensor‐based tractography results for the CST were oversimplifying the bundles, failing to capture its fanning into the lateral primary sensory‐motor cortex in most cases. While this indicates that automated tractography could be safely used in the presurgical setting, leading to a reduction of processing/work time, and eliminate user bias, manual CSD FT might yet prove in future studies to be superior to automated CSD FT. Parcellation‐based tractography solutions remain vulnerable to errors rooted in parcellation failure, which is being actively addressed by approaches based on direct segmentation of the white matter bundles, such as TractSeg (Wasserthal et al., 2018) and their benefits in the presurgical setting remain to be explored as well.

The main limitation of this study was the small sample size, which motivated not including covariates for number of dMRI shells, EPI distortion correction, pathology type, nor the approach used for intraoperative mapping (awake or not), as well as not including DES current characteristics such as polarity, amplitude, nor pulse widths in our analysis. However, the two‐part linear model accounted for these factors by allowing for random intercepts and slopes per patient. Additionally, we did not include perioperative patient status in this analysis. However, potential differences due to number of shells acquired and EPI distortion correction methods were explored and found to be relatively minor, see Table S8. Lastly, while deterministic CSD tractography was not included in this study, its performance was evaluated by applying tckgen using SD_Stream (Tournier et al., 2012) with and without anatomical constraints, see Figure S5. Deterministic CSD FT did not perform as well as probabilistic CSD FT did, but slightly better than probabilistic tensor‐based FT.

In summary, both exploratory analyses and statistical modeling for the CST indicate that CSD tractograms are more accurate than DTI in this sample. Specifically, CSD showed a higher rate of true positives and a slight increase in false positives, but there were no significant differences in true or false negatives. While no statistical tests were conducted for the AFs due to their limited number (*N* = 7), they seemed to behave qualitatively similar to the CST. These findings demonstrate the superior accuracy of CSD‐based MRI tractography techniques over DTI‐based methods among preoperative neurosurgical patients in this study. Although the results are consistent within this group and could be expected to hold in larger samples, extrapolating them to a wider population warrants caution. Hence, while this study offers valuable insights for similar clinical contexts, its relevance to diverse patient groups, various neurological conditions, or different MRI technologies requires further exploration.

## 5 CONCLUSIONS

In this study of preoperative neurosurgical patients, CSD‐based tractography demonstrated superior accuracy over DTI‐based methods when evaluated against intraoperative DES stimulation. Specifically, CSD tractograms were closer to DES coordinates, exhibited higher sensitivity, and were more likely to overlap with positive sensory‐motor functional response locations. While both manual and automated DTI tractography methods showed comparable performance, CSD's superiority underscores the need for further exploration of advanced tractography methods like CSD. Although these findings are consistent within the studied group, caution is advised when extrapolating to a broader population due to the study's small sample size. Future research should consider diverse patient groups, various neurological conditions, and different MRI technologies to validate and expand upon these conclusions.

## CONFLICT OF INTEREST STATEMENT

The authors report no competing interests.

## CLINICAL TRIAL REGISTRATION

The manuscript is part of a cross‐sectional observational clinical study registered on clinicaltrials.gov with the identifier NCT06040580.

## Supporting information


**APPENDIX S1.** Supporting information.

## Data Availability

The data that support the findings of this study are available on request from the corresponding author. The data are not publicly available due to privacy or ethical restrictions.
