# Supplementary material for: Comparative validation of automated presurgical tractography based on constrained spherical deconvolution and diffusion tensor imaging with direct electrical stimulation
Source: Hum Brain Mapp. 2024 Apr 22;45(6):e26662. doi: 10.1002/hbm.26662 (PMC11033921; doi:10.1002/hbm.26662)
Supplement: Supplementary file 1 — APPENDIX S1. Supporting information. [file HBM-45-e26662-s001.docx]

**Comparative Validation of Automated Presurgical Tractography based on Constrained Spherical Deconvolution and Diffusion Tensor imaging with Direct Electrical Stimulation**

**Supplementary material**

**Tractogram filtering details**

In addition to the methods previously described for KUL_FWT we also used template-based tractogram filtering with RecoBundles(Garyfallidis et al., 2018) as implemented in Scilpy (Bore et al., 2015/2021) (scil_recognize_single_bundle.py) with model clustering threshold = 4 and pruning threshold = 8. Patient tractograms were warped to the template space prior to template-based filtering by applying ANTs generated warps and transforms to the bundle using MRtrix3.

The anatomical constraints used in our FWT (Radwan et al., 2022) bundle-specific tractography framework, while similar, are distinct from the anatomically-constrained tractography (ACT) approach implemented in the tckgen -act option (Smith et al., 2012) in MRtrix3, which is tailored for whole-brain tractography. We have developed an alternative approach that adapts ACT principles for bundle-specific tracking within FWT. This approach involves constraining the spatial extent of seeding and inclusion VOIs to a two-pass dilated version of the grey-white matter interface and excludes all CSF voxels from the mask used with tckgen.

**Two-part linear model details**

The first part (A) used a logit-link for binary response (distance=0 vs. distance >0) and a generalized linear mixed model to predict probability of nonoverlap (distance > 0), and the second part (B) used a log-normal linear mixed model for the distance measures (distance >0) between nonoverlapping tractogram-DES coordinate pairs.

The thresholded distance measures were used as the dependent variable and DES response type (positive and negative), tractography methods (FACT, TP, ATP, iFOD2, and AiFOD2) were used as predictors in both parts of the model. Both models were adjusted for bundle to TIV and lesion to TIV ratios, which were used as covariates, and DES response type x tractography methods interactions were considered but were omitted as they were not significant.

For ease of interpretation, we discuss and plot the probability of overlap (distance = 0) for the first part (A), and log distances (B) from the second part are back-transformed to distance in mm.

Results were interpreted in the following context: Predicted probability of overlap (distance < cutoff) with pDES coordinates was analogous to true-positive rate. Predicted probability of overlap with nDES coordinates was analogous to false-positive rate. Predicted distances to non-overlapping (distance > cutoff) nDES coordinates were analogous to true-negative rate, and predicted distances to non-overlapping pDES coordinates were analogous to false-negative rate.

In our analysis, missing data was represented and handled by assigning 'NAN' (Not a Number) values. The decision to use 'NAN' ensured that the missing data did not influence the statistical calculations or skew the results. The analysis was then carried out on the remaining dataset, excluding the 'NAN' values.

| Supplementary table 1: Detailed demographics, pathological type, distribution, and volumes | | | | | |
| --- | --- | --- | --- | --- | --- |
| **Patients** | **Age**  **(years)** | **Sex** | **Lesion type - WHO grade** | **Lesion side and lobe** | **Lesion volume (ml)** |
| **PT001** | 60 – 65 | M | Glioblastoma – IV | R - Fronto-parietal | 66.81 |
| **PT002** | 5 – 10 | F | FCD (Type I) | L - Frontal | 1.20 |
| **PT003** | 35 – 40 | M | Transitional type meningioma – I | L - Frontal | 54.08 |
| **PT004** | 30 – 35 | F | Oligodendroglioma – II | L - Fronto-parietal | 124.78 |
| **PT005** | 70 – 75 | M | Glioblastoma – IV | R - Parieto-occipital | 32.38 |
| **PT006** | 40 – 45 | F | Glioblastoma – IV | R - Temporo-fronto-parietal | 217.08 |
| **PT007** | 65 – 70 | F | Glioblastoma – IV | R - Frontal | 26.78 |
| **PT008** | 35 – 40 | M | Glioblastoma – IV | L - Temporo-fronto-parietal | 88.92 |
| **PT009** | 10 – 15 | M | FCD (Type IIB) | L - Frontal | 15.67 |
| **PT010** | 60 – 65 | M | Glioblastoma – IV | R - Fronto-parietal | 93.83 |
| **PT011** | 40 – 45 | M | Multifocal astrocytoma – II | R - Fronto-temporo-parieto-occipital | 123.29 |
| **PT012** | 60 – 65 | F | Oligodendroglioma – II | L - Frontal | 53.33 |
| **PT013** | 45 – 50 | F | FCD (Type IIB) | L - Parietal | 5.22 |
| **PT014** | 30 – 35 | M | Oligodendroglioma – III | R - Frontal | 92.15 |
| **PT015** | 35 – 40 | M | Glioblastoma – IV | R - Frontal | 232.13 |
| **PT016** | 30 – 35 | F | Oligodendroglioma – II | L - Parietal | 22.55 |
| **PT017** | 60 – 65 | M | Glioblastoma – IV | R - Parietal | 42.52 |
| **PT018** | 30 – 35 | F | Oligodendroglioma – III | L - Frontal | 46.89 |
| **PT019** | 55 – 60 | M | Astrocytoma – III | L - Fronto-temporal | 40.21 |
| **PT020** | 55 – 60 | M | Oligodendroglioma – II | L - Frontal | 11.53 |
| **PT021** | 15 – 20 | M | FCD (Type IIB) | R - Frontal | 4.81 |
| **PT022** | 10 – 15 | M | DNET – I | L - Frontal | 34.21 |
| PT = patient, M = male, F = female, FCD = focal cortical dysplasia, DNET = dysembryoplastic neuroepithelial tumor, WHO = world health organization, R = right, L = left, ml = milliliters, IQR = interquartile range | | | | | |

| Supplementary Table 2: MRI acquisition parameters | | | | |
| --- | --- | --- | --- | --- |
|  | **dMRI - part1** | | **dMRI - part2** | **dMRI - part3** |
| **Pulse sequence / acquisition plane** | 2D spin-echo EPI / Axial | | | |
| **TR/TE ms / FA °** | 4500/85 / 85 | | | |
| **Voxel size in mm^3** | 1.96*1.96*2.00 | | | |
| **Acquisition matrix** | 112*112*69 | | | |
| **In-plane / through plane acceleration** | SENSE 1.6 / Multiband 3 | | | |
| **Pixel BW** | 2997 | | | |
| **Phase encoding - Fat shift directions** | AP - A | | | AP - P |
| **Bvalue (****s/mm^2)** | 1200 | 2500 | | 0 |
| **Number of diffusion directions** | 127 | 124 | | 0 |
| **Number of non-diffusion volumes** | 1 | 4 - 9 | | 4 - 7 |
| dMRI = diffusion magnetic resonance imaging, EPI = echo planar imaging, TR = repetition time, TE = echo time, ms = milliseconds, FA = flip angle, SENSE = SENSitivity encoding in plane parallel imaging acceleration, BW = bandwidth, PE = phase encoding, AP = anteroposterior, A = anterior, P = posterior | | | | |

| **Supplementary table 3: Direct electrical stimulation (DES) mapping and dMRI and tractography details** | | | | | |
| --- | --- | --- | --- | --- | --- |
| **Patients** | **Surgery:**  **Awake**  –  **DES +/-ve** | **Positive DES effect** | **Stim. Thr.**  **Ctx/SubCtx** | **dMRI data**  **shells / rev.phase** | **Bundles** |
| **PT001** | Y – 1/6 | Motor dysarthria | 20 / -mA | 2 / RP-B0 | AF + CST |
| **PT002** | N – 4/6 | Motor right leg, foot, wrist, and hand | 6 / 4 mA | 2 / RP-B0 | CST |
| **PT003** | Y – 3/3 | Motor right leg, foot, and hand | 4 mA | 2 / RP-B0 | CST |
| **PT004** | Y – 1/7 | Motor face, right hand, and arm | - / 5 mA | b2500 / RP-B0 | AF + CST |
| **PT005** | Y – 2/4 | Motor left hand | 16 / 5 mA | 2 / RP-B0 | CST |
| **PT006** | Y – 2/8 | Sensory-Motor left leg | 20 / 5 mA | 2 / RP-B0 | CST |
| **PT007** | Y – 2/6 | Sensory-motor left leg | 16 / 10 mA | 2 / RP-B0 | CST |
| **PT008** | Y – 2/5 | Difficulty finding words, and sensory mouth | 20 / 2 mA | b1200 / - | AF + CST |
| **PT009** | Y – 2/4 | Speech arrest, paraphasia, and motor face | 8 / - mA | 2 / RP-B0 | AF + CST |
| **PT010** | N – 3/0 | Motor left leg | Not recorded | 2 / RP-B0 | CST |
| **PT011** | Y – 0/6 | None | - | 2 / RP-B0 | CST |
| **PT012** | Y – 0/7 | None | - | b1200 / RP-B0 | AF |
| **PT013** | Y – 7/0 | Sensory-motor right arm and hand | 10 / 5 mA | 2 / RP-B0 | CST |
| **PT014** | Y – 2/6 | Motor left hand and foot | 12 / - mA | 2 / RP-B0 | CST |
| **PT015** | N – 2/0 | Motor left hand | - / 10 mA | 2 / RP-B0 | CST |
| **PT016** | Y – 3/6 | Motor right hand | 12 / 5 mA | b1200 / RP-B0 | CST |
| **PT017** | Y – 2/2 | Motor left foot, leg, hand and arm | 4 / 12 mA | 2 / RP-B0 | CST |
| **PT018** | Y – 3/8 | Motor right hand, lips, and dysarthria | 20 / 5 mA | b1200 / - | CST |
| **PT019** | Y – 1/6 | Motor right hand | 20 / 10 mA | 2 / RP-B0 | CST |
| **PT020** | Y – 1/4 | Motor right arm | - / 10 mA | b1200 / RP-B0 | AF + CST |
| **PT021** | Y – 3/6 | Motor left wrist and hand | 4 mA | 2 / RP-B0 | CST |
| **PT022** | Y – 0/8 | None | - | 2 / RP-B0 | AF |
| PT = patient, DES = direct electrical stimulation, +ve = positive DES, -ve = negative DES, dMRI = diffusion magnetic resonance imaging, AF = arcuate fasciculus, CST = corticospinal tract, Y = awake surgery, N = general anaesthesia with motor and somatosensory evoked potentials (MEP/SSEP), Ctx = cortical, SubCtx= subcortical, mA = milliampere, RP-B0 = reversed-phase non-diffusion weighted spin-echo EPI volume. | | | | | |

**Supplementary figure 1 Arcuate fasciculi (AF) representative images** using all methods in lateral projection. The FACT tractogram outputs shown in green are generated from volume rendered voxel masks with the T1-brain image silhouette shown underneath. The other 4 methods TP, ATP, iFOD2, and AiFOD2 are shown as 3D rendered streamlines with end-point directional color coding. PT = patient, FACT = fiber assignment by continuous tracking, TP tensor probabilistic, ATP = anatomically constrained tensor probabilistic, iFOD2 = probabilistic tractography by second order integration over spherical harmonics, AiFOD2 = anatomically constrained iFOD2

### Tractogram similarity analysis results

DSC and JI scores were used to evaluate the similarity of tractograms across different FT methods. As expected, we found higher similarity between AiFOD2 and iFOD2 than between TP, ATP and iFOD2 bundles. However, clinical DTI FACT tractograms also showed higher DSC and JI compared to TP and ATP, see **S.table 6** for summarized descriptive statistics of tractogram similarity measures. Tractogram shape similarity analysis using DSC and JI with iFOD2 tractograms as the reference showed the expected pattern of mild to moderate similarity between DTI and iFOD2 tractograms. This also showed that the CSD-based and DTI-based tractograms were not identical among themselves, confirming the differences noted visually.

| Supplementary table 4: Summarized descriptive statistics for bundle similarity measures for all methods compared to iFOD2 | | | | | | | | |
| --- | --- | --- | --- | --- | --- | --- | --- | --- |
| **Tractography methods** | **FACT** | | **TP** | | **ATP** | | **AiFOD2** | |
| **Measures** | **JI** | **DSC** | **JI** | **DSC** | **JI** | **DSC** | **JI** | **DSC** |
| **max** | 0.41 | 0.59 | 0.34 | 0.51 | 0.42 | 0.59 | 0.76 | 0.86 |
| **min** | 0.10 | 0.18 | 0.13 | 0.23 | 0.06 | 0.11 | 0.51 | 0.68 |
| **mean** | 0.26 | 0.41 | 0.24 | 0.39 | 0.20 | 0.32 | 0.66 | 0.80 |
| **median** | 0.25 | 0.39 | 0.24 | 0.39 | 0.20 | 0.33 | 0.69 | 0.82 |
| **StD** | 0.09 | 0.11 | 0.06 | 0.08 | 0.08 | 0.11 | 0.07 | 0.05 |
| **IQR** | 0.08 | 0.10 | 0.06 | 0.08 | 0.04 | 0.06 | 0.02 | 0.02 |
| iFOD2 = second order integration over fiber orientation distributions, FACT = fiber assignment by continuous tracking, TP = tensor probabilistic, ATP = anatomically constrained TP, AiFOD2= anatomically constrained iFOD2, JI = Jaccard index, DSC = Dice similarity coefficient, StD = standard deviation, IQR = interquartile range | | | | | | | | |
|  |  |  |  |  |  |  |  |  |

| Supplementary table 5: Descriptive statistics for distance measures (in mm) per tractography method | | | | | | | | | | |
| --- | --- | --- | --- | --- | --- | --- | --- | --- | --- | --- |
| **DES type** | **Bundle** | **FT** | **Mean** | **StD** | **Median** | **Q1** | **Q3** | **IQR** | **Range** | **N** |
| **pDES** | **CST** | **FACT** | 12.37 | 11.73 | 8 | 2.25 | 21 | 18.75 | 0 - 39 | 46 |
|  |  | **TP** | 13.14 | 11.76 | 8.50 | 3.75 | 22 | 18.25 | 0 - 39 | 44 |
|  |  | **ATP** | 14.78 | 11.45 | 10.50 | 5.25 | 23 | 17.75 | 0 - 39 | 46 |
|  |  | **iFOD2** | 5.22 | 6.75 | 2 | 1 | 6 | 5 | 0 - 34 | 46 |
|  |  | **AiFOD2** | 4.89 | 6.06 | 3 | 1 | 6 | 5 | 0 - 27 | 46 |
| **nDES** |  | **FACT** | 27.24 | 12.47 | 28 | 19 | 37 | 18 | 3 - 57 | 93 |
|  |  | **TP** | 27.37 | 12.18 | 29 | 19.50 | 37 | 17.50 | 1 - 54 | 85 |
|  |  | **ATP** | 30.23 | 12.16 | 32 | 22.50 | 39 | 16.50 | 3 - 58 | 93 |
|  |  | **iFOD2** | 15.68 | 10.79 | 13 | 6.50 | 22 | 15.50 | 0 - 43 | 93 |
|  |  | **AiFOD2** | 15.78 | 10.71 | 13 | 8 | 21 | 13 | 0 - 48 | 93 |
| **pDES** | **AF** | **FACT** | 12.80 | 6.18 | 13 | 12 | 17 | 5 | 3 - 19 | 5 |
|  |  | **TP** | 9.40 | 3.85 | 10 | 6 | 12 | 6 | 5 - 14 | 5 |
|  |  | **ATP** | 8.60 | 3.58 | 8 | 6 | 10 | 4 | 5 - 14 | 5 |
|  |  | **iFOD2** | 5.60 | 5.41 | 3 | 2 | 8 | 6 | 1 - 14 | 5 |
|  |  | **AiFOD2** | 5.20 | 4.82 | 4 | 2 | 8 | 6 | 0 - 12 | 5 |
| **nDES** |  | **FACT** | 15 | 10.71 | 12 | 6.25 | 21.75 | 15.50 | 0 - 40 | 30 |
|  |  | **TP** | 13.90 | 11.26 | 13.50 | 3.50 | 21 | 17.50 | 0 - 42 | 30 |
|  |  | **ATP** | 14 | 11.84 | 11.50 | 4.25 | 20.50 | 16.25 | 0 - 42 | 30 |
|  |  | **iFOD2** | 9.37 | 7.79 | 8.50 | 2.75 | 12.75 | 10 | 0 - 33 | 30 |
|  |  | **AiFOD2** | 9.67 | 8.25 | 8 | 3.50 | 13.50 | 10 | 0 - 35 | 30 |
| DES = direct electrical stimulation, pDES = positive DES, nDES = negative DES, FT = fiber tractography method, StDev = standard deviation, Q = quartile, IQR = interquartile range, N = number, FACT = fiber assignment by continuous tracking, TP = tensor probabilistic, ATP = anatomically constrained TP, iFOD2 = probabilistic tractography by second-order integration over spherical harmonics, AiFOD2 = anatomically constrained iFOD2 | | | | | | | | | | |

**Supplementary figure 2:** ROC plots for raw distance measures pooled for all methods (black), DTI (blue), and CSD (red), showing the optimal distance cutoff, and area under the curve (AUC), and comparisons in bottom right panel. FACT = fiber assignment by continuous tracking, TP tensor probabilistic, ATP = anatomically constrained tensor probabilistic, iFOD2 = probabilistic tractography by second order integration over spherical harmonics, AiFOD2 = anatomically constrained iFOD2

### Supplementary figure 3: ROC plots for each tractography method using raw distances on the left and using averaged distances per patient for nDES and pDES separately on the right. Also shown are the optimal distance cutoffs, and area under the curve (AUC), and all methods ROCs compared in bottom right panel. FACT = fiber assignment by continuous tracking, TP tensor probabilistic, ATP = anatomically constrained tensor probabilistic, iFOD2 = probabilistic tractography by second order integration over spherical harmonics, AiFOD2 = anatomically constrained iFOD2

| **Supplementary table 6: Summarized results of DeLong pairwise tests comparing the ROC curves from averaged distance measures** | | | | | | |
| --- | --- | --- | --- | --- | --- | --- |
| Pairwise comparisons | **Estimate1** | **Estimate2** | **Statistic** | **p.value** | **CI.low** | **CI.high** |
| FACT v TP | 67.46 | 64.15 | 0.62 | 0.54 | -0.07 | 0.14 |
| FACT v ATP | 67.46 | 68.18 | -0.22 | 0.82 | -0.07 | 0.06 |
| FACT v iFOD2 | 67.46 | 77.38 | -1.45 | 0.15 | -0.23 | 0.03 |
| FACT v AiFOD2 | 67.46 | 77.69 | -1.55 | 0.12 | -0.23 | 0.03 |
| TP v ATP | 64.15 | 68.18 | -0.81 | 0.42 | -0.14 | 0.06 |
| TP v iFOD2 | 64.15 | 77.38 | -1.84 | 0.07 | -0.27 | 0.01 |
| TP v AiFOD2 | 64.15 | 77.69 | -1.92 | 0.05 | -0.27 | 0.00 |
| ATP v iFOD2 | 68.18 | 77.38 | -1.37 | 0.17 | -0.22 | 0.04 |
| ATP v AiFOD2 | 68.18 | 77.69 | -1.46 | 0.15 | -0.22 | 0.03 |
| iFOD2 v AiFOD2 | 77.38 | 77.69 | -0.21 | 0.84 | -0.03 | 0.03 |
| ROC = receiver operating characteristic, CI = confidence interval, DES = direct electrical stimulation, pDES = positive DES, nDES = negative DES, FACT = fiber assignment by continuous tracking, TP = tensor probabilistic, ATP = anatomically constrained TP, iFOD2 = probabilistic tractography by second-order integration over spherical harmonics, AiFOD2 = anatomically constrained iFOD2 | | | | | | |

| Supplementary table 7: Results of post hoc tests after two-part linear modelling for the CST at 10.5 mm distance cut-off | | | | | | | | |
| --- | --- | --- | --- | --- | --- | --- | --- | --- |
| **Tractography methods** | **Model part A** | | | | **Model part B** | | | |
|  | **T** | **CI low/high** | **P_uncorr_** | **P_FWE_** | **T** | **CI low/high** | **P_uncorr_** | **P_FWE_** |
| **FACT v TP** | -1.07 | -1.514/0.490 | 0.298 | 0.595 | -1.28 | -0.152/0.0371 | 0.218 | 0.537 |
| **FACT v ATP** | -2.30 | -2.129/-0.094 | 0.034 | 0.102 | -2.48 | -0.199/-0.0163 | 0.023 | 0.070 |
| **FACT v iFOD2** | **5.01** | **1.281/3.130** | **<0.001** | **<0.001** | **7.19** | **0.252/0.460** | **<0.001** | **<0.001** |
| **FACT v AiFOD2** | **4.98** | **1.263/3.109** | **<0.001** | **<0.001** | **7.26** | **0.256/0.464** | **<0.001** | **<0.001** |
| **TP v ATP** | -1.26 | -1.602/0.403 | 0.225 | 0.595 | -1.14 | -0.143/0.0422 | 0.269 | 0.537 |
| **TP v iFOD2** | **5.94** | **1.756/3.678** | **<0.001** | **<0.001** | **8.24** | **0.308/0.519** | **<0.001** | **<0.001** |
| **TP v AiFOD2** | **5.93** | **1.741/3.655** | **<0.001** | **<0.001** | **8.33** | **0.312/0.522** | **<0.001** | **<0.001** |
| **ATP v iFOD2** | **6.85** | **2.300/4.333** | **<0.001** | **<0.001** | **9.44** | **0.360/0.566** | **<0.001** | **<0.001** |
| **ATP v AiFOD2** | **6.84** | **2.284/4.311** | **<0.001** | **<0.001** | **9.54** | **0.364/0.570** | **<0.001** | **<0.001** |
| **IFOD2 v AiFOD2** | -0.06 | -0.748/0.709 | 0.956 | 0. 956 | -0.07 | -0.109/0.117 | 0.941 | 0.941 |
| AiFOD2 = anatomically constrained iFOD2, ATP = anatomically constrained Tensor Probabilistic, CI = confidence interval, CST = corticospinal tract, FACT = fiber assignment by continuous tracking, iFOD2 = second order integration over fiber orientation distributions, P_uncorr_ = uncorrected p values, P_FWE_ = Hochberg family-wise error rate corrected p value, T = t-statistic, TP = tensor probabilistic. Bold values denote significant differences on P_FWE_ | | | | | | | | |

Due to the limited sample size, more covariates e.g., for acquisition strategy, MR machine used, specific b-values, couldn’t be included in our main analyses. However, in order to explore the potential impact of the number of shells and EPI distortion correction stratgey, we compared mean and median distances for single- and multi-shell data, and for data with and without reversed-phase B0s (topup v synB0-DISCO), see S.table 8. Minor differences were found between these subsamples. Notably, since FACT always used only 1 shell without any EPI distortion correction, the differences it captures are caused solely by inter-subject differences. The differences detected between the other tractography methods did not exceed that captured by FACT. Further, the differences in distances that could be ascribed to differences in number of shells acquired and/or EPI distortion correction approach (presence of absence of a reversed phase image), were estimated by subtracting the difference of each method from that of FACT. The results show that inter-subject differences were larger than the differences due to shell number or EPI distortion correction approach.

| Supplementary table 8: Estimated differences in average and median distance measures (in mm) due to differences in number of shells (a) and EPI distortion correction approach (b) | | | | | | | | | | |
| --- | --- | --- | --- | --- | --- | --- | --- | --- | --- | --- |
| (a) | **Absolute differences between single and multishell data for pDES** | | | | | **Absolute differences between single and multishell data for nDES** | | | | |
|  | **FACT** | **TP** | **ATP** | **iFOD2** | **AiFOD2** | **FACT** | **TP** | **ATP** | **iFOD2** | **AiFOD2** |
| AVERAGE | **7.1** | 6.7 | 5.4 | 0.5 | 0.3 | **4** | 4.3 | 1.6 | 0.5 | 0.6 |
| MEDIAN | **11** | 8.5 | 6.5 | 1 | 1 | **5.5** | 7.5 | 5 | 1 | 0 |
|  | **Estimated differences due to number of shells for pDES** | | | | | **Estimated differences due to number of shells for nDES** | | | | |
| AVERAGE |  | 0.4 | 1.7 | 6.6 | 6.8 |  | 0.3 | 2.4 | 3.5 | 3.4 |
| MEDIAN |  | 2.5 | 4.5 | 10 | 10 |  | 2 | 0.5 | 4.5 | 5.5 |
| (b) | **Absolute differences between data with and without reversed phase B0s for pDES** | | | | | **Absolute differences between data with and without reversed phase B0s for nDES** | | | | |
|  | **FACT** | **TP** | **ATP** | **iFOD2** | **AiFOD2** | **FACT** | **TP** | **ATP** | **iFOD2** | **AiFOD2** |
| AVERAGE | **4.5** | 3.5 | 2.3 | 4.3 | 3.9 | **1.2** | 1.4 | 0.3 | 4.1 | 4 |
| MEDIAN | **9** | 1.5 | 0 | 1 | 2 | **1.5** | 4 | 0.5 | 5 | 3.5 |
|  | **Estimated differences due to presence of absence of reversed phase B0s for pDES** | | | | | **Estimated differences due to presence of absence of reversed phase B0s for nDES** | | | | |
| AVERAGE |  | 1 | 2.2 | 0.2 | 0.6 |  | 0.2 | 0.9 | 2.9 | 2.8 |
| MEDIAN |  | 7.5 | 9 | 8 | 7 |  | 2.5 | 1 | 3.5 | 2 |

**
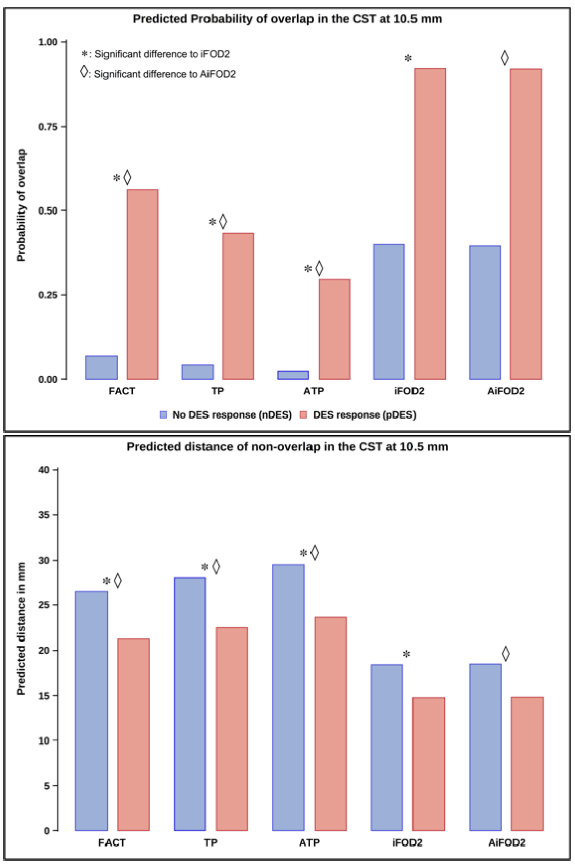
**

**Supplementary figure 4:** Bar plots for predicted probability of overlap between the CST and DES coordinates (Top) and predicted distances to nonoverlapping DES coordinates (Bottom) at 10.5 mm distance cutoff. CSD methods showed significantly higher probability of overlap, and lower distance if not overlapping compared to DTI methods. Differences between nDES and pDES were comparable between FT methods. CST = corticospinal tract, FACT = fiber assignment by continuous tracking, TP = tensor probabilistic, ATP = anatomically constrained TP, iFOD2 = probabilistic tractography by second order integration over spherical harmonics, AiFOD2 = anatomically constrained iFOD2.

**
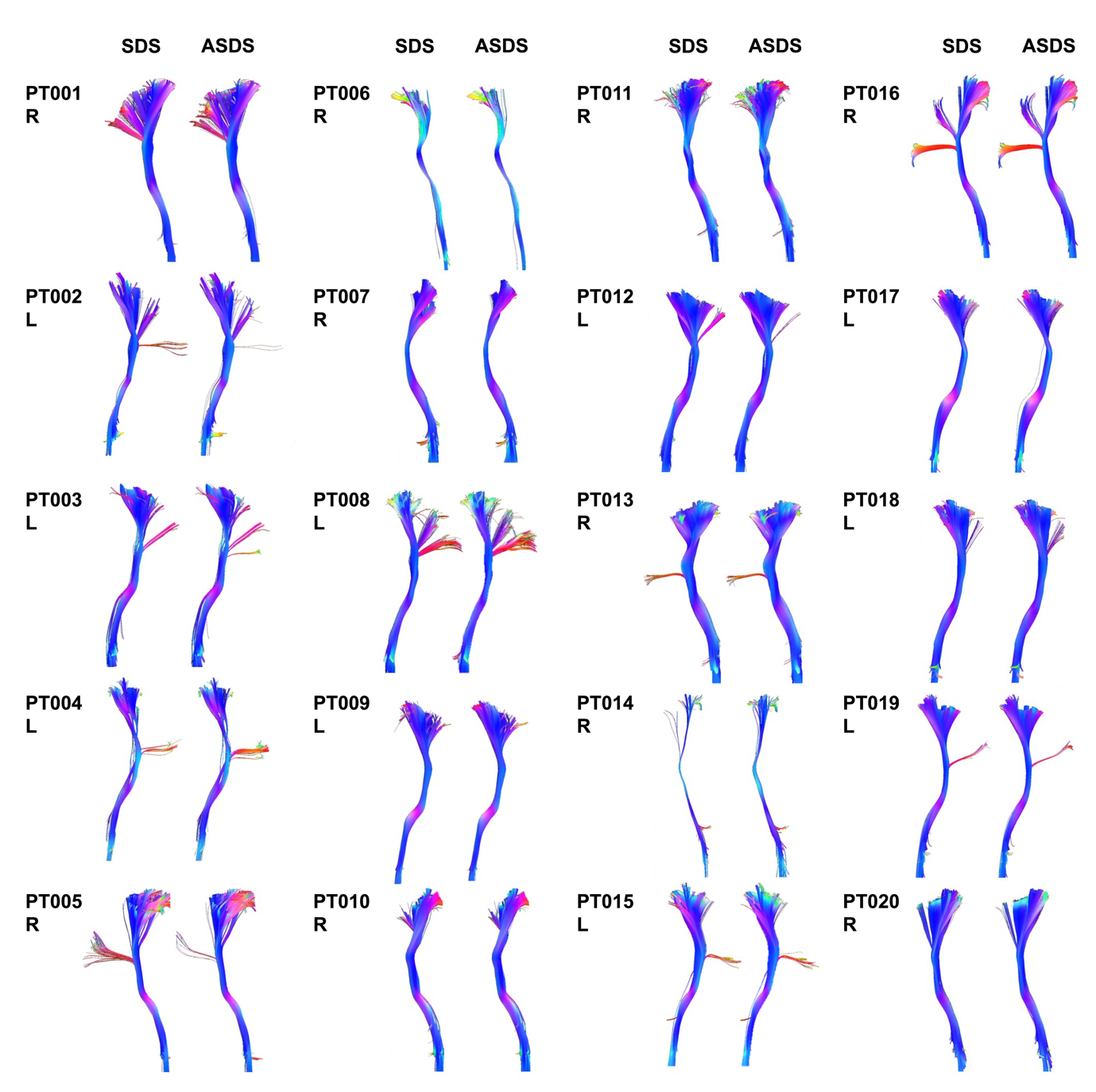
**

**Supplementary figure 5:** Anterior views of the CST tractograms for each patient reconstructed with deterministic CSD tractography in MRtrix3 SD_Stream (SDS), and anatomically-constrained SD_Stream (ASDS), PT = patient, R = right, L = Left.

# Supplementary references

1. Bore, A., Rheault, F., Theaud, G., & Théberge, A. (2021, July 28). *Scilpy*. https://github.com/scilus/scilpy (Original work published 2015)
2. Brett, M., Markiewicz, C. J., Hanke, M., Côté, M.-A., Cipollini, B., McCarthy, P., Jarecka, D., Cheng, C. P., Halchenko, Y. O., Cottaar, M., Larson, E., Ghosh, S., Wassermann, D., Gerhard, S., Lee, G. R., Wang, H.-T., Kastman, E., Kaczmarzyk, J., Guidotti, R., … freec84. (2022). *nipy/nibabel: 3.2.2* [Computer software]. Zenodo. https://doi.org/10.5281/zenodo.6617121
3. Duffau, H., Capelle, L., Sichez, J., Faillot, T., Abdennour, L., Law Koune, J. D., Dadoun, S., Bitar, A., Arthuis, F., Van Effenterre, R., & Fohanno, D. (1999). Intra-operative direct electrical stimulations of the central nervous system: The Salpêtrière experience with 60 patients. *Acta Neurochirurgica*, *141*(11), 1157–1167. https://doi.org/10.1007/s007010050413
4. Garyfallidis, E., Côté, M.-A., Rheault, F., Sidhu, J., Hau, J., Petit, L., Fortin, D., Cunanne, S., & Descoteaux, M. (2018). Recognition of white matter bundles using local and global streamline-based registration and clustering. *NeuroImage*, *170*, 283–295. https://doi.org/10.1016/j.neuroimage.2017.07.015
5. Harris, C. R., Millman, K. J., van der Walt, S. J., Gommers, R., Virtanen, P., Cournapeau, D., Wieser, E., Taylor, J., Berg, S., Smith, N. J., Kern, R., Picus, M., Hoyer, S., van Kerkwijk, M. H., Brett, M., Haldane, A., del Río, J. F., Wiebe, M., Peterson, P., … Oliphant, T. E. (2020). Array programming with NumPy. *Nature*, *585*(7825), Article 7825. https://doi.org/10.1038/s41586-020-2649-2
6. Radwan, A. M., Sunaert, S., Schilling, K., Descoteaux, M., Landman, B. A., Vandenbulcke, M., Theys, T., Dupont, P., & Emsell, L. (2022). An atlas of white matter anatomy, its variability, and reproducibility based on constrained spherical deconvolution of diffusion MRI. *NeuroImage*, *254*, 119029. https://doi.org/10.1016/j.neuroimage.2022.119029
7. Smith, R. E., Tournier, J.-D., Calamante, F., & Connelly, A. (2012). Anatomically-constrained tractography: Improved diffusion MRI streamlines tractography through effective use of anatomical information. *NeuroImage*, *62*(3), 1924–1938. https://doi.org/10.1016/J.NEUROIMAGE.2012.06.005
8. Virtanen, P., Gommers, R., Oliphant, T. E., Haberland, M., Reddy, T., Cournapeau, D., Burovski, E., Peterson, P., Weckesser, W., Bright, J., van der Walt, S. J., Brett, M., Wilson, J., Millman, K. J., Mayorov, N., Nelson, A. R. J., Jones, E., Kern, R., Larson, E., … van Mulbregt, P. (2020). SciPy 1.0: Fundamental algorithms for scientific computing in Python. *Nature Methods*, *17*(3), Article 3. https://doi.org/10.1038/s41592-019-0686-2
9. Zangaladze, A., Sharan, A., Evans, J., Wyeth, D. H., Wyeth, E. G., Tracy, J. I., Chervoneva, I., & Sperling, M. R. (2008). The effectiveness of low-frequency stimulation for mapping cortical function. *Epilepsia*, *49*(3), 481–487. https://doi.org/10.1111/j.1528-1167.2007.01307.x
